# Genome-wide identification of the *AlkB* homologs gene family, *PagALKBH9B* and *PagALKBH10B* regulated salt stress response in *Populus*

**DOI:** 10.3389/fpls.2022.994154

**Published:** 2022-09-20

**Authors:** Ye Zhao, Qi Guo, Sen Cao, Yanting Tian, Kunjin Han, Yuhan Sun, Juan Li, Qingshan Yang, Qingju Ji, Ronald Sederoff, Yun Li

**Affiliations:** ^1^Key Laboratory of Genetics and Breeding in Forest Trees and Ornamental Plants of Ministry of Education, College of Biological Sciences and Technology, National Engineering Research Center of Tree Breeding and Ecological Restoration, Engineering Technology Research Center of Black Locust of National Forestry and Grassland Administration, Beijing Forestry University, Beijing, China; ^2^Natural Resources and Planning Bureau of Yanshan County, Cangzhou, Hebei, China; ^3^Shandong Academy of Forestry, Jinan, Shandong, China; ^4^Cangzhou Municipal Forestry Seeding and Cutting Management Center, Cangzhou, China; ^5^Forest Biotechnology Group, Department of Forestry and Environmental Resources, North Carolina State University, Raleigh, NC, United States

**Keywords:** AlkB homologs, poplar 84K, m^6^A RNA methylation, demethylase, salt

## Abstract

The AlkB homologs (*ALKBH*) gene family regulates N^6^-methyladenosine (m^6^A) RNA methylation and is involved in plant growth and the abiotic stress response. Poplar is an important model plant for studying perennial woody plants. Poplars typically have a long juvenile period of 7–10 years, requiring long periods of time for studies of flowering or mature wood properties. Consequently, functional studies of the *ALKBH* genes in *Populus* species have been limited. Based on *AtALKBHs* sequence similarity with *Arabidopsis thaliana*, 23 *PagALKBHs* were identified in the genome of the poplar 84K hybrid genotype (*P. alba*  ×  *P. tremula* var. *glandulosa*), and gene structures and conserved domains were confirmed between homologs. The PagALKBH proteins were classified into six groups based on conserved sequence compared with human, Arabidopsis, maize, rice, wheat, tomato, barley, and grape. All homologs of *PagALKBHs* were tissue-specific; most were highly expressed in leaves. ALKBH9B and ALKBH10B are m^6^A demethylases and overexpression of their homologs *PagALKBH9B* and *PagALKBH10B* reduced m^6^A RNA methylation in transgenic lines. The number of adventitious roots and the biomass accumulation of transgenic lines decreased compared with WT. Therefore, *PagALKBH9B* and *PagALKBH10B* mediate m^6^A RNA demethylation and play a regulatory role in poplar growth and development. Overexpression of *PagALKBH9B* and *PagALKBH10B* can reduce the accumulation of H_2_O_2_ and oxidative damage by increasing the activities of SOD, POD, and CAT, and enhancing protection for Chl a/b, thereby increasing the salt tolerance of transgenic lines. However, overexpression lines were more sensitive to drought stress due to reduced proline content. This research revealed comprehensive information about the *PagALKBH* gene family and their roles in growth and development and responsing to salt stress of poplar.

## Introduction

AlkB homologs (ALKBH) are a family of specific demethylases which belong to the dioxygenase superfamily and depend upon Fe^2+^ and α-ketoglutarate to catalyze demethylation of diverse substrates such as proteins, unique nucleic acids (tRNA, snRNA, bubbled DNA, bulged DNA, ds/ss DNA, and mRNA) (Trewick et al., 2002; Xu et al., 2020). About 40 years ago (Kataoka et al., 1983), the *Escherichia. coli* AlkB (EcAlkB) protein was found to repair damage from alkylating agents by dealkylation. While *E. coli* has only a single gene encoding ALKB, animals and plants have a family of homologs (ALKBH) (Wei et al., 1996; Kurowski et al., 2003; Chen et al., 2019; Wakisaka et al., 2019; Liang et al., 2020; Marcinkowski et al., 2020; Cai et al., 2021).

As a type of demethylase, the ALKBH gene family was proposed to be an eraser for epigenetic modification. Some N^6^-methyladenosine (m^6^A) RNA demethylases have been identified in Arabidopsis such as *ALKBH9B* (Martínez-Pérez et al., 2017), *ALKBH10B* (Duan et al., 2018), and *SLALKBH2* in tomato (Zhou et al., 2019). In *Arabidopsis thaliana*, ALKBH10B, an m^6^A RNA demethylase can demethylate m^6^A-based mRNA modifications and mediate the m^6^A demethylation of transcripts of the key flowering time genes *flowering locus T* (*FT*), *squamosa promoter binding protein-like 3* (*SPL3*), and *SPL9* to specifically regulate the floral transition process (Duan et al., 2018). In addition, karrikins (a group of plant regulators present in smoke after burning) and cesium (Nelson et al., 2009) can induce the up-regulation of *ALKBH10B* (Hampton et al., 2004; Nelson et al., 2009, 2010). The expression of *ALKBH10B* decreased in the Arabidopsis *dpa1* mutant, in which the γ subunit of the plastid ATP synthase has been inactivated (Bosco et al., 2004). *ALKBH10B*-mediated m^6^A demethylation affects the transcriptional level of stress response genes and regulates seed germination, seedling growth, and survival of *Arabidopsis thaliana* in response to salt stress or ABA (Shoaib et al., 2021). *SlALKBH2* bind to the mRNA of the DNA demethylase gene *SlDML2* in tomatoes and regulates its m^6^A modification and stability for fruit maturation (Zhou et al., 2019). *ALKBH9*-mediated m^6^A methylation may be a regulatory strategy to control cytoplasmic-replicating RNA viruses. ALKBH9B accumulated in cytoplasmic granules, colocalized with siRNA bodies, and was associated with P bodies and acted in mRNA silencing and/or mRNA decay (Martínez-Pérez et al., 2017). In *Arabidopsis*, the demethylation activity of *AtALKBH9B* positively regulates the infectivity of alfalfa mosaic virus (AMV), inhibition of *AtALKBH9B* increased the relative abundance of m^6^A in the AMV genome and inhibited systemic invasion of plants (Martínez-Pérez et al., 2017). The AtALKBH6 protein can bind to both m^6^A-labeled and m^5^C-labeled RNAs in *Arabidopsis thaliana* and an *Atalkbh6* mutant showed a much lower survival rate than the wild-type under salt, drought, or heat stress, the transcript levels of ABA signaling-related genes may be down-regulated in the *Atalkbh6* mutant (Huong et al., 2020). The role of *ALKBH1D* as a demethylase in chloroplasts may be similar to that of human *ALKBH1* in mitochondria, where it is essential for tRNA biogenesis (Kawarada et al., 2017). *ALKBH8* may be involved in the mcm5U and 5-methoxycarbonylmethyl-2′-O-methyluridine-2′-O-ribose methylated derivative process in *Arabidopsis* (Leihne et al., 2011; Endres et al., 2015). In *ALKBH8B*-overexpressing transgenic plants, global m^6^A levels were decreased and higher salt-tolerant phenotypes were exhibited compared to wild-type (Huong et al., 2022). Salt stress may induce the expression of ALKBH9 in roots, while high temperature inhibits the expression of *ALKBH10A* (Ma et al., 2006; Merret et al., 2015).

Soil salinization is a long-term abiotic stressor in extreme environments, causing huge losses to agriculture and forestry production (Janz and Polle, 2012; Zhao et al., 2020). Such extreme environmental conditions can affect the growth and development of plants (Hatfield and Prueger, 2015; Yu et al., 2016), weakening them so that they are more susceptible to disease or insect attacks. Salt stress causes osmotic stress, ion toxicity, and reactive oxygen species damage, resulting in blocked respiration, nutrient deficiency, decreased photosynthesis and transpiration, and inhibited growth and development (Tester and Davenport, 2003; Yu et al., 2016). Plants have evolved complex mechanisms to respond to salt stress, including osmotic regulation, ion balancing, the oxidative stress response, signal transduction *via* osmotic and ionic homeostasis signaling pathways, organelle stress responses, hormonal changes, and gene expression regulation (Zhao et al., 2020).

Poplar is an important woody model in plant molecular biology and it is also an important economic genus of forest trees (Bradshaw et al., 2000). However, the functions of the ALKBH family in *Populus* are not yet adequately defined, especially in response to abiotic stresses. Poplar 84K (*Populus alba* × *Populus tremula* var. *glandulosa*) is a well-known hybrid between species of white poplar (Qiu et al., 2019). The completion of the *Populus* genome sequence has allowed us to comprehensively characterize the *ALKBH* gene family in poplar (Tuskan et al., 2006; Ma et al., 2014; Qiu et al., 2019; Hofmeister et al., 2020; An et al., 2021). Here, we identified 23 *PagALKBHs* from poplar 84K, compared the collinearity, amino acid sequence, gene structure, and conserved motifs among homologs, and then classified them into six groups based on the conserved sequence. We described the expression patterns of the *PagALKBHs* in different tissues. Transgenic experiments showed that *PagALKBH9B* and *PagALKBH10B* are m^6^A RNA demethylases in poplar and that they inhibit adventitious root formation and biomass accumulation. Overexpression of *PagALKBH9B* and *PagALKBH10B* can reduce the accumulation of H_2_O_2_ and oxidative damage by increasing the activities of SOD, POD, and CAT, and increase the content of proline to reduce osmotic stress, thereby increasing the salt tolerance of transgenic lines. These results provide a molecular basis for further research on the adaptation and evolution of the ALKBH family in *Populus*.

## Materials and methods

### Plant material and growth conditions

The poplar 84K (*Populus alba* × *Populus tremula* var. *glandulosa*, a hybrid white poplar, hybrid female parent is *P. alba* and the hybrid male parent is *P. glandulosa*) plantlets were propagated by tissue culture, about 3 cm shoot tips were cut and placed in rooting medium (1/2 MS + 0.01 mg/l NAA + 0.1 mg/l IBA + 30 g/l sucrose +6.5 g/l agar) as described in the previous study (Zhao et al., 2022). The subculture plantlets were placed in 25°C culture chamber with a photoperiod of 16 h light (12,000 lux) and relative humidity of approximately 70% for 4 weeks and transferred to Hoagland’s nutrient solution for 1 week. The hydroponic plantlets were treated with 100 mM NaCl for 0, 6, 12, 24, and 48 h, and leaves were collected for detection of *PagALKBH9B* and *PagALKBH10B* expression abundance. In order to detect the tissue expression specificity of *PagALKBHs*, different tissues and organs were collected and immediately frozen in liquid nitrogen, and then stored at −80°C. At least six plantlets were pooled together as a biological replicate and three replicates were used for each experiment.

Poplar (*Populus tomentosa*) was used for genetic transformation as described earlier (Du et al., 2012). The adventitious roots of different transgenic lines growing in a rooting medium for 10 days were collected to detect the expression abundance of adventitious root (AR) regulatory genes. For phenotypic evaluation and the analysis of physiological indexes, transgenic plants and WT were grown in the rooting medium for 3 weeks for observation. For phenotypic observation and the analysis of physiological indexes, the 3 weeks old WT and transgenic lines were taken out from the medium and transplanted into mixed soil (perlite: vermiculite = 1: 3) for 2 weeks. Different lines were treated with a certain concentration (0, 100, and 200 mM) of salt solution and observed continuously. After 3 days of treatment, the second and third leaves fully stretched from top to bottom were collected for physiological indexes detection. The responses of each line to drought stress were compared under natural drought conditions, the second and third leaves fully stretched from top to bottom were collected for physiological indexes detection after 9 days. At least six plantlets were pooled together as biological replicates and three replicates were used to count the growth indexes and phenotypic observation.

### Identification of *PagALKBH* genes and synteny analysis

Chromosome-level genomes of poplar 84K (*Populus alba* × *P. tremula* var. *glandulosa*) were referenced as published (Qiu et al., 2019). The genomes of Arabidopsis (*Arabidopsis thaliana*), maize (*Zea mays*), and rice (*Oryza sativa*) were downloaded from the Ensemblm^1^ and Phytozome database.^2^ We searched the poplar 84K genome with the ALKBH protein sequences of Arabidopsis and rice and selected all sequences with an *e*-value <= 1*e*-10 and then used Pfam^3^ and SMART tools^4^ to determine whether each candidate 2OG-Fe (II) and Fe^2+^ domains, identified *ALKBH* family genes (Letunic et al., 2009). Collinear blocks were identified by *PagALKBHs* duplication events in the MCScanX (Wang et al., 2012). We integrated and plotted the data using Circos (Krzywinski et al., 2009). DNAMAN 6.0 was used to align amino acid sequences (Nong et al., 2019).

### Phylogenetic analysis, gene structure, and protein motifs

The ALKBH protein sequences of different species were imported into MEGA 7.0 software to construct a phylogenetic tree by neighbor-joining (NJ). A bootstrap value was set to 1,000 replicates (Gascuel, 2006; Tamura et al., 2013) and visualized using Evolview (Zhang et al., 2012). The Gene Structure Display Server (GSDS)^5^ was used to recognize the exons and introns of *PagALKBH* genes (Zhang et al., 2012). The conserved motifs of PagALKBH proteins were identified using MEME Suite Web server^6^ (Bailey et al., 2009) and the maximum number of motifs was specified as 10. TBtools was used to visualize gene structure and conserved domains (Chen et al., 2020).

### RNA isolation and reverse transcription-quantitative real-time polymerase chain reaction analysis

Total RNA was isolated using TRIzol reagent (TransGen Biotech, Beijing, China). The PrimeScript™ RT kit with gDNA eraser was then used to reverse-transcribe the total RNA into cDNA (TaKaRa, Beijing, China) and detected by 1% agarose gel electrophoresis, qualified cDNA is diluted with different concentrations according to the Ct value of the pre-experiment. Reverse transcription-quantitative real-time polymerase chain reaction (RT-qPCR) was performed with the SYBR Green master mix (TaKaRa). All primers used were detected by PCR and listed in Supplementary Table S1. The 2^–ΔΔCt^ method was used to calculate the relative abundance of *PagALKBH* transcripts (Livak and Schmittgen, 2001) with the reference gene *PagActin* as an internal standard (Wang et al., 2021). Three biological replicates with three technical replicates were performed for each sample.

### Vector construction, poplar transformation, and characterization

The primers *PagALKBH9B*-F, *PagALKBH9B*-R, and *PagALKBH10B*-F, *PagALKBH10B*-R were used to amplify the nucleotide sequences PagALKBH9B and PagALKBH10B, respectively. The PagALKBH9B and PagALKBH10B were subcloned into the pBI121 vector under the control of the CaMV35S promoter. Agrobacterium-mediated stable transformation was performed as previously described (Du et al., 2012). In sterile conditions, take 4–6 weeks of *P. tomentosa* tissue culture plantlets leaves, cut the main vein with a knife, and placed in the OD_600_ = 0.4 *Agrobacterium* infection for 10 min. The leaves were taken out, dried with sterile filter paper, and the back of leaves was laid down on the differentiation culture medium (MS + 1.0 mg/l 6-BA +0.2 mg/l NAA + 80 μM acetosyringone), and cultured at 25°C in dark for 2 days. After co-culture, the explants were suctioned with sterile filter paper to remove the suction residual bacteria liquid, transferred to the screening medium (differentiation medium +250 mg/l cephalosporin +30 mg/l kanamycin), light cycle 16 h/8 h, 25°C, every 10 days to replace the selection medium. The length of equal transformed buds was about 2 cm, cut and inserted into rooting medium (1/2 MS + 0.2 mg/l NAA + 0.2 mg/l IBA + 250 mg/l cephalosporin +40 mg/l Kana). Rooted kanamycin-resistant plantlets were screened based on the fusion fragment of the CaMV35S promoter, *PagALKBH9B,* and *PagALKBH10B* using genomic PCR amplification with the forward primer 35S-F and reverse primers *PagALKBH9B*-R and *PagALKBH10B*-R. Then, the transgenic plants were analyzed using RT-qPCR with the primers *PagALKBH9B*-qF, *PagALKBH9B*-qR, and *PagALKBH10B*-qF, *PagALKBH10B*-qR. Primers used are listed in Supplementary Table S1.

### Determination of the m^6^A RNA methylation level

Total RNA was isolated from leaves of transgenic lines and WT as previously described using the TRIzol reagent (TransGenBiotech, Beijing, China). RNA quality was assessed using a NanoDrop 2000 spectrophotometer (Thermo Scientific, Waltham, MA, United States) and agarose gel electrophoresis. Oligo (dT) magnetic beads were used to capture the mRNA, and liquid washing was used to remove the non-mRNA, so as to obtain the complete mRNA in the total RNA (TIANGEN, Beijing, China). The m^6^A level was determined with an Aderr kit (Epigentek, Farmington, NY, United States). In this assay, RNA is bound to strip wells using RNA high binding solution. m^6^A is detected using capture and detection antibodies. The detected signal is enhanced and then quantified by reading the absorbance in a microplate spectrophotometer. The amount of m^6^A is proportional to the OD intensity measured. m^6^A (%) = (Sample OD-NC OD)/S/(PC OD-NC OD)/P*100% (NC: negative control; PC: positive control; S: RNA sample inputs, ng; P: positive control inputs, ng).

### Physiological indices analysis

After 3 days of salt stress at different concentrations, the leaves were rounded with a diameter of 1 cm and immersed in 3,3′-diaminobenzidine (DAB) solution (1 mg/m L, 50 mM Tris–HCl, pH 3.8) at 28°C in the dark for 16 h. Then, incubate with 95% ethanol at 70°C for 5 min to remove chlorophyll. After cooling, the leaves were transferred to fresh ethanol at room temperature and photographed. The H_2_O_2_ content of each line was detected according to the H_2_O_2_ content detection kit (NJJCbio, China) following the manufacturer’s instructions. The MDA and proline contents in the fresh leaves were measured spectrophotometrically according to Zhao et al. (2022). The leaf discs were punched with a 1 cm diameter puncher and placed in a clean test tube. After adding 5 ml deionized water containing Tween 20, initial (S1) and final (S2) conductivity were measured after sealing boiling water bath for 8 min and cooling balance for 3 min. At the same time, the blank conductivity (S0) of distilled water was measured. Electrolyte leakage rate (%) = (S1-S0)*100/(S2-S0). The contents of Chl and Chl b and antioxidant enzyme activity levels of SOD, POD, and CAT were evaluated using the corresponding assay kits (NJJCbio, Nanjing, China).

### m^6^A-IP-qPCR

m^6^A-IP-qPCR was carried out as previously described with minor modifications (Zhou et al., 2021). Total RNA extracted was preheated at 75°C for 5 min, and then cooled immediately on ice. 200 μg total RNA was fragmented into 300 nt short fragments using RNA fragmentation buffer (10 mM Tris–HCl, pH 7.0, 10 mM ZnCl_2_) at 95°C for 3 min, and then 50 mM EDTA was added to terminate the fragmentation reaction and then cooled immediately on ice. Add 2.5 times volume of alcohol, 0.1 times volume of 3 M sodium acetate (pH 5.3), 100 μg/ml glycogen, overnight at −80°C. 15,000 *g* centrifugal 20 min, precipitation using pre-cooling 75% alcohol wash twice, 15,000 *g* centrifugal 10 min, and ventilation cabinet drying 5 min. Then resuspension with 100 μl DEPC-treated water. Reservation of 2 μg total RNA as input and incubation of 100 μg total RNA with 2–5 μg of anti-m^6^A polyclonal antibody (Synaptic Systems, 202003, Germany) at 4°C for 2 h. The washed immunomagnetic beads protein A was added to the RNA solution and incubated at 4°C for 2 h. The antibody bound to the protein beads and the RNA complex were collected by the magnetic force frame, and washed twice with 1 × IP buffer, high salt buffer (50 mM Tris–HCl, 1 M NaCl, 1 mM EDTA, 1% NP-40, and 0.1% SDS), respectively. 200 μl elution buffer (6.7 mM m^6^A in 1 × IP buffer, 300 U mL^–1^ RNase inhibitor) was added to the mixture for 2 h with continuous shaking at 4°C. After RNA was precipitated by ethanol, centrifuged at 15,000 g for 20 min at 4°C, washed twice with 75% ethanol, centrifuged at 15,000 g for 5 min at 4°C, then dried in the ventilating cabinet for 5 min, and resuspended with 10 μl RNase-free water. Then, both the m^6^A-containing total RNA and the input total RNA were submitted to RT-qPCR and primers listed in Supplementary Table S1. The 2^–ΔΔCt^ method was used to calculate relative m^6^A enrichment. Three biological replicates with three technical replicates were performed for each sample.

### Statistical analysis

All results were expressed as the mean ± standard error (SE) in triplicate or more. Statistical analyses were performed using SPSS 22.0 software (IBM, Armonk, NY, United States), and significant differences in the measured parameters were inferred according to Duncan’s multiple range test. Significant differences between the two groups of data were evaluated for comparisons (*p* < 0.05).

## Results

### Genome-wide identification of *PagALKBHs*

**Figure 1:**
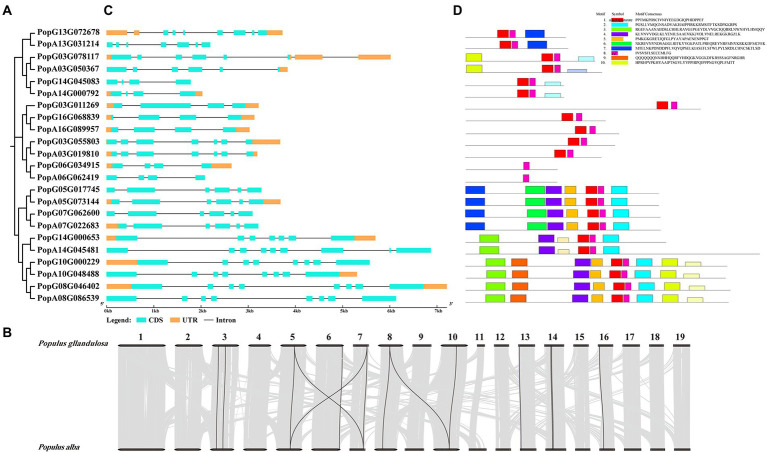
**(A)** Phylogeny of PagALKBH proteins identified in poplar 84K. **(B)** Genome distribution and collinearity of the *PagALKBH* family in poplar 84K. Black lines indicate the collinear relationships of *PagALKBHs*, gray lines represent other than *PagALKBHs* collinear relationships. **(C)** Gene structures of *PagALKBHs*. The exons, untranslated regions (UTR), and introns were represented by amber and cyan rectangles, and the blank line, respectively. **(D)** Conserved motifs of *PagALKBHs*. Different motifs are represented by different colors.

To identify all the ALKBH members in poplar 84K (*Populus alba* × *Populus tremula* var. *glandulosa*), 14 Arabidopsis and 9 rice ALKBH amino acid sequences were used as queries (Yue et al., 2019) and then screened against Pfam and SMART databases. Twenty-three *PagALKBHs* were identified in poplar 84K, among them, 11 PagALKBHs were present in the female hybrid parent (*P. alba*), and 12 PagALKBHs were present in the male hybrid parent (*P. glandulosa*). There was a difference in the number of *ALKBH* genes among hybrid parents, hybrid female parent lost one located on chromosome 3 (Figure 1A; Supplementary Table S2). Further sequence alignment revealed high sequence similarity of homologs from hybrid parents of poplar 84K (Supplementary Figure S1). Then we used Circos to draw whole-genome synteny blocks and studied the whole-genome for duplication (WGD) events in the *PagALKBH* gene family among hybrid parents of poplar 84K (Figure 1B). Collinearity was conserved for 14 pairs of genes among hybrid parents and most homologous genes have a one-to-one correspondence across species.

### Gene structure and conserved motifs of *PagALKBHs*

As shown in Figure 1C, similar exon and intron structures exist between genes that are evolutionarily closely related, there were identical intron and exon structures between homologs except *PopG14G000653* and *PopA14G045481*, *PopA14G045481* had two extra exons. On the contrary, there were differences in the untranslated region (UTR) between homologs between different hybrid parents. Except for the homologous genes PopG16G068839 and PopA16G089957 which contain the same UTR structure, most of the other homologous genes that exist in pairs only have one of them. Different from gene structure, conserved sequences were highly consistent among homologs. In addition, genes close to each other on the evolutionary tree often contain the same conserved domains and similar motifs suggesting conservation in function of PagALKBH family proteins, and unique motifs may predict specific biological functions. Among the PagALKBHs, motif 8 harboring the Fe^2+^ binding site was conserved, which was characteristic of the ALKBH gene family. However, another conserved domain of the ALKBH gene family, α-ketoglutarate, was missing in *PopG06G034915* and *PopA06G062419*, which may indicate differences in function.

### Phylogenetic analysis of *PagALKBHs*

**Figure 2:**
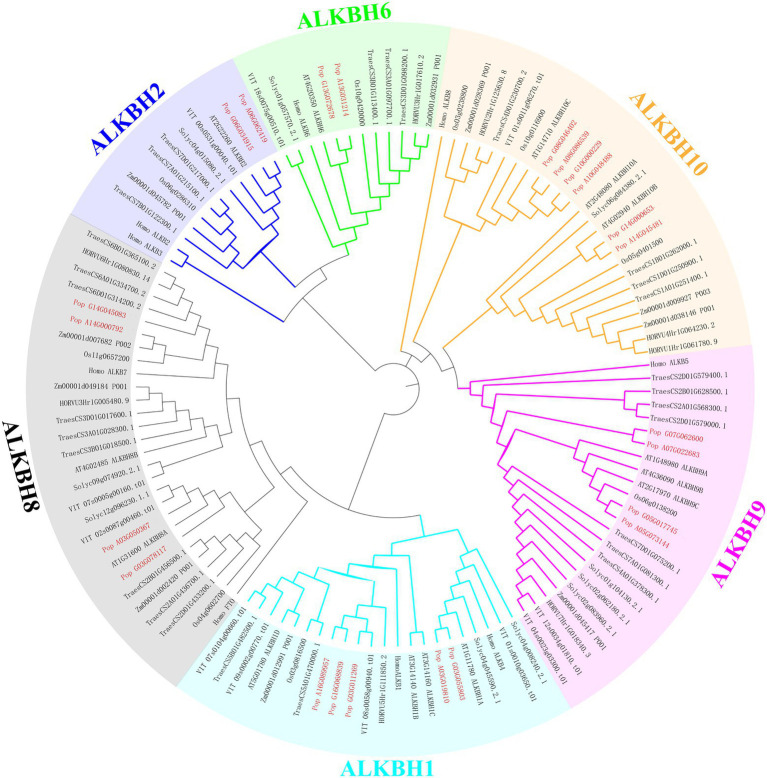
Phylogeny of ALKBH proteins. Phylogenetic relationships of human (*Homo sapiens*) Arabidopsis (*A. thaliana*), maize (*Zea mays*), rice (*Oryza sativa*), wheat (*Triticum aestivum*) tomato (*Solanum lycopersicum*) barley (*Hordeum vulgare*) grape (*Vitis vinifera*), and poplar 84K (*Populus alba*  ×  *P. tremula* var. *glandulosa*) were estimated using maximum likelihood. Different colors represent different groups.

To demonstrate the relationships between species, and to illustrate their evolution, a phylogenetic tree was constructed among human (*Homo sapiens*), Arabidopsis (*A. thaliana*), maize (*Zea mays*), rice (*Oryza sativa*), wheat (*Triticum aestivum*), tomato (*Solanum lycopersicum*), barley (*Hordeum vulgare*), grape (*Vitis vinifera*), and poplar 84 K (*Populus alba* × *P. tremula* var. *glandulosa*; Figure 2). The proteins clustered into six groups (ALKBH1, ALKBH2, ALKBH6, ALKBH8, ALKBH9, ALKBH10) following earlier nomenclature (Marcinkowski et al., 2020). ALKBHs showed differences in numbers within species. Of all ALKBH proteins, the largest amount of ALKBHs was found in wheat, while the lowest was found in barley which lost the member of the ALKBH2 group. The ALKBH10 group was the most abundant group, with 15 members that displayed differences in number between different species, ALKBH2 and ALKBH6 had the smallest number of members. The ALKBH9 and ALKBH10 groups are relatively closely related to the human m^6^A RNA demethylation modification enzyme ALKBH5, while another demethylase FTO, although classified in the ALKBH8 group, is farther related to shuffling.

### Tissue-specific expression of the *PagALKBH* genes in poplar 84K

**Figure 3:**
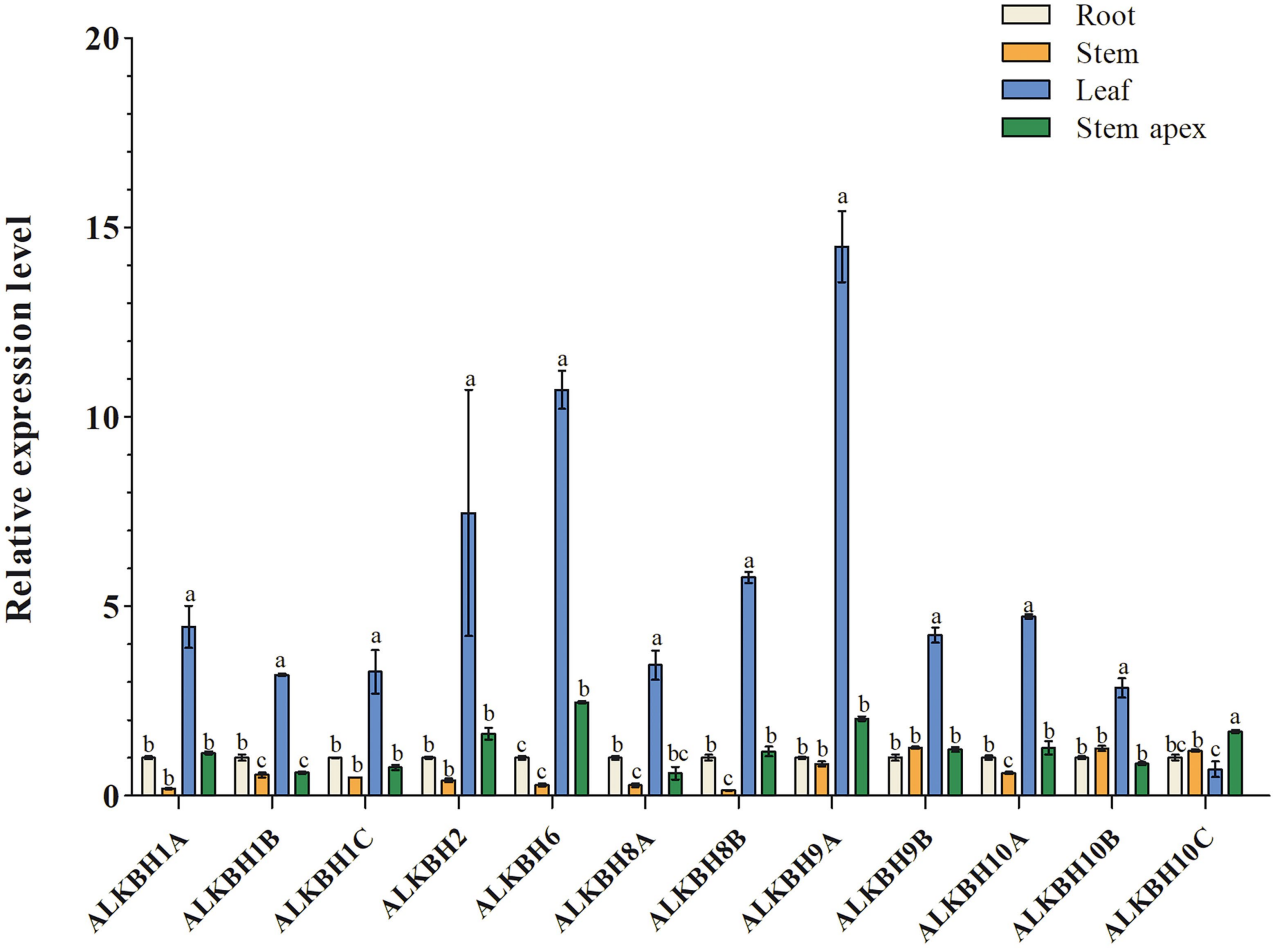
Tissue-specific expression patterns of *PagALKBHs* poplar 84K. The roots, stems, leaves, and stem apex of 84K poplars under normal growth conditions were collected, different tissues of at least six 84K poplar were collected as a replicate. The data represented averages ± SE of three replicates. The different letter indicated significant differences between different tissue.

To explore the biological functions of *PagALKBH* genes, RT-qPCR was used to survey the relative abundance of *PagALKBH* transcripts in different tissues (root, stem, leaf, and stem apex). The relative abundance of *PagALKBH* transcripts can be determined for various tissues and organs of poplar to identify tissue-specific differences (Figure 3). All the *PagALKBHs* had similar expression patterns where the transcript level in leaves was relatively high and the abundance in other tissues was low, with the exception of ALKBH10C, which was lowest in leaves.

### Overexpression of *PagALKBH9B* and *PagALKBH10B* reduced m^6^A RNA methylation

**Figure 4:**
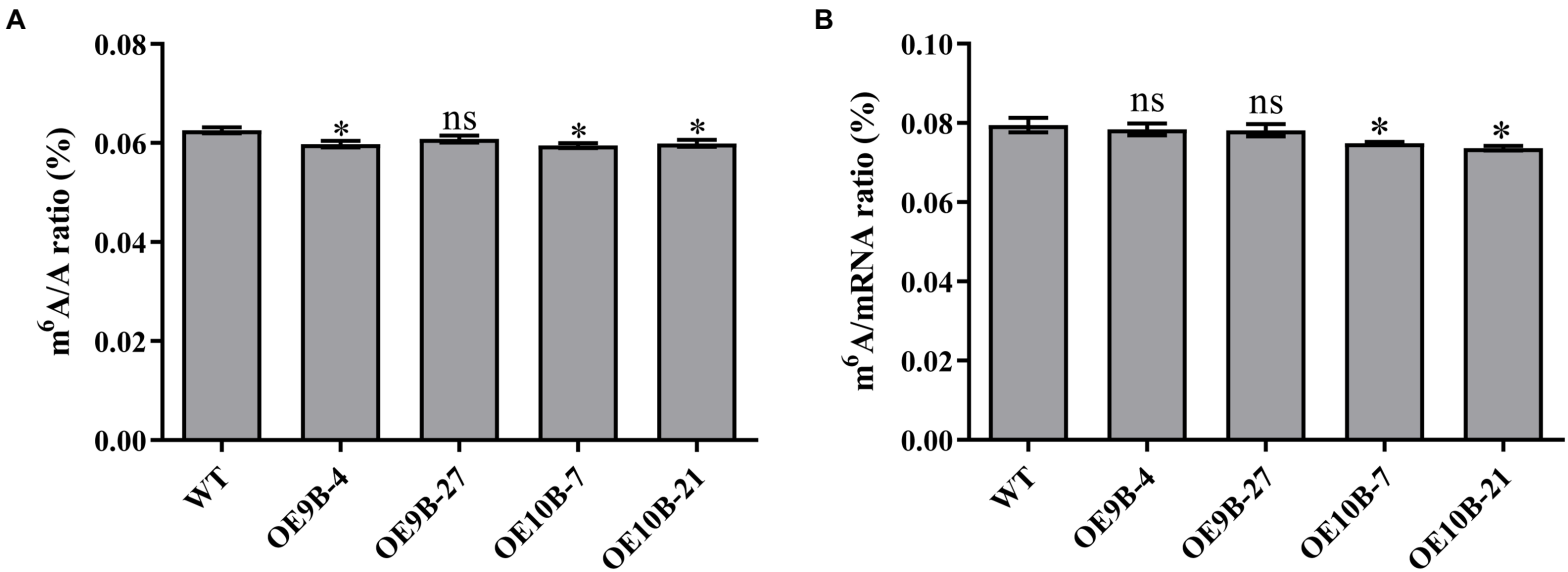
N^6^-methyladenosine (m^6^A) RNA methylation level. **(A)** Global N^6^-methyladenosine (m^6^A) RNA methylation level in WT and transgenic lines under normal conditions. **(B)** N^6^-methyladenosine (m^6^A) RNA methylation level on mRNA in WT and transgenic lines under normal conditions. At least six 84K poplars were collected as a replicate. The asterisks indicated significant differences between WT and transgenic plants (*p  <  0.05).

ALKBH9B and ALKBH10B were m^6^A RNA methylation demethylases, and overexpression of *ALKBH9B* and *ALKBH10B* reduces the level of m^6^A RNA methylation in Arabidopsis (Martínez-Pérez et al., 2017; Duan et al., 2018). To determine whether *PagALKBH9B* and *PagALKBH10B* regulate m^6^A methylation in poplar, transgenic poplar harboring the *PagALKBH9B* and *PagALKBH10B* genes under the control of CaMV35S promoter (*35S::PagALKBH9B* and *35S::PagALKBH10B*) were generated by *Agrobacterium*-mediated transformation. After kanamycin screening, genomic PCR identification, and RT-qPCR analysis, then, four transgenic lines (OE9B-4 and OE9B-27 overexpressing of *PagALKBH9B* and OE10B-7 and OE10B-21 overexpressing of *PagALKBH10B*), were used for further analyses (Supplementary Figure S2). By detecting the overall level of m^6^A in the leaves of WT and transgenic lines under normal growth conditions, it was found that overexpression of *PagALKBH9B* and *PagALKBH10B* reduced the m^6^A RNA modification level on total RNA in transgenic poplars (Figure 4A) and overexpression of *PagALKBH9B* did not significantly affect m^6^A modification on mRNA (Figure 4B).

### Overexpressing of *PagALKBH9B* and *PagALKBH10B* inhibited adventitious root formation in poplar 84K

**Figure 5:**
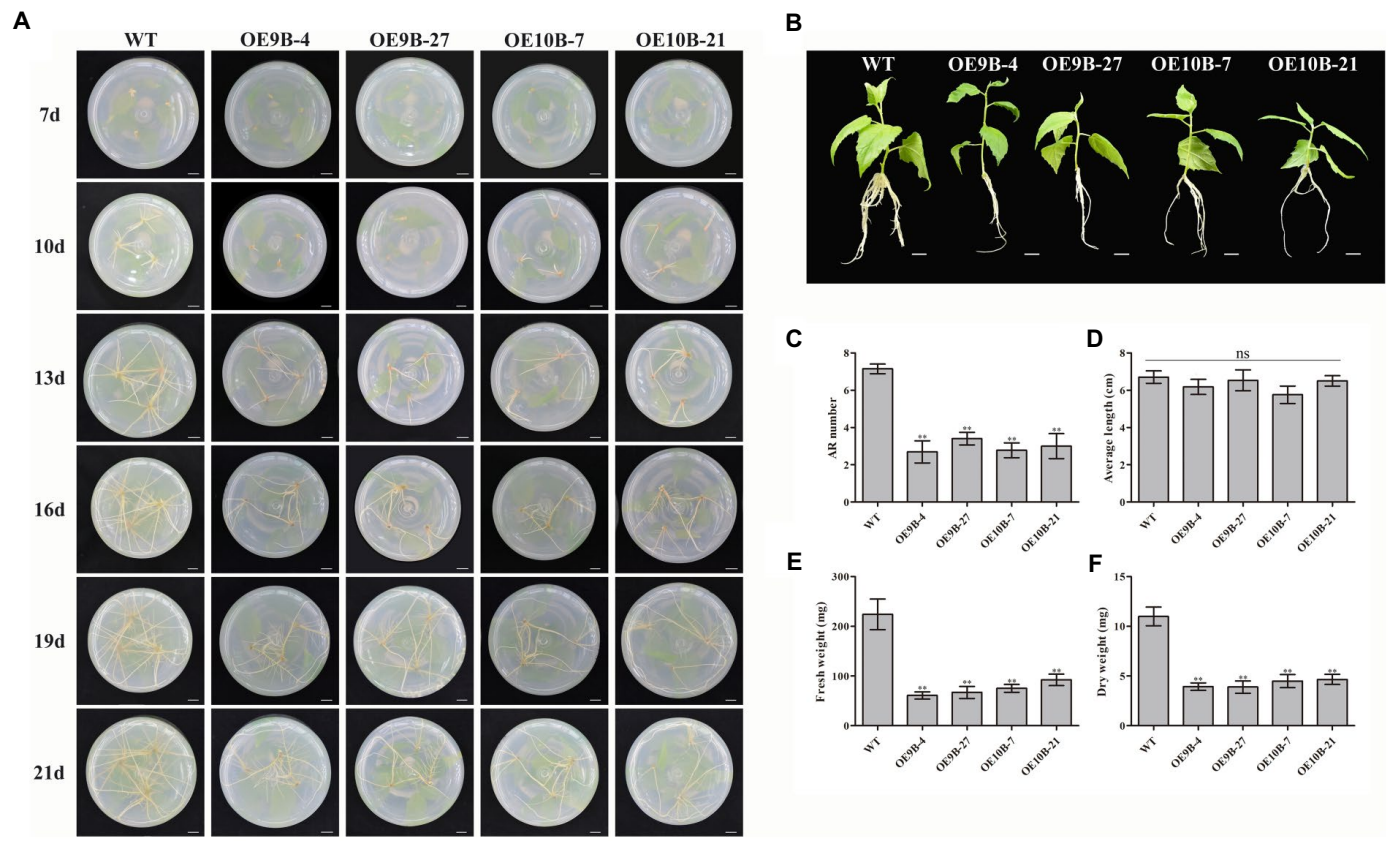
Phenotypes of transgenic lines and WT cuttings 3 weeks after establishment in rooting medium. **(A)** The adventitious roots of transgenic lines and WT formed and developed over time. Bar = 1 cm. **(B)** Images of cuttings from different lines 3 weeks after establishment. Bar = 1 cm. **(C)** AR number between different lines. **(D)** Average length of ARs between different lines. **(E)** Fresh weight of ARs between different lines. **(F)** Dry weight of ARs between different lines. The experiments were performed three times and the similar results were obtained, at least six 84K poplars were collected as a replicate. The bars represent means ± SE (*n* = 6) and the asterisks indicate significant differences between WT and transgenic plants (*p < 0.05, **p < 0.01).

To determine whether *PagALKBH9B* and *PagALKBH10B* participated in ARs formation, we compared the AR phenotypic changes of overexpressing transgenic plantlets and WT plantlets under normal conditions (Figure 5A). When the stem segments were inserted into the rooting medium for 7 days, WT had formed adventitious roots, while all transgenic lines did not. After 13 days, although all transgenic lines had formed ARs, the number of ARs was significantly less than that of WT, which continued to 21 days. After culture in rooting medium for 21 days, we took out different lines for phenotype observation and growth index statistical analysis. The observation of the ARs phenotype and the statistics of ARs number showed that the number of ARs of transgenic lines overexpressing of *PagALKBH9B* and *PagALKBH10B* was significantly less than that of WT, which was consistent with the observations in tissue culture (Figures 5B,C). There was no significant difference in the average length of ARs among different lines (Figure 5D). Compared with WT, the biomass of ARs (fresh weight and dry weight) in transgenic lines decreased significantly (Figures 5E,F). The expression of *ARF6*, *ARF8*, *GH3.5*, *YUCCA6,* and *ABCB19* that have been provedto regulate the formation of ARs was inhibited in transgenic lines overexpressing *PagALKBH9B* and *PagALKBH10B* (Li, 2021), and *IAA28* was inhibited in transgenic lines overexpressing *PagALKBH10B* (Supplementary Figure S3A). However, we found that the m^6^A levels of these genes were not significantly different in transgenic lines, and they may not be directly regulated by *PagALKBH9B* or *PagALKBH10B* (Supplementary Figure S3B). Overexpression of *PagALKBH9B* did not reduce the plant height of transgenic lines, while the plant height of transgenic lines overexpressing *PagALKBH10B* was significantly inhibited compared with WT, although the biomass accumulation between transgenic lines was similar and less compared with WT (Supplementary Figure S4). It was therefore reasonable to infer that *PagALKBH9B* and *PagALKBH10B* regulate the demethylation of common genes and specific targets involved in ARs formation and plant growth.

### Overexpressing of *PagALKBH9B* and *PagALKBH10B* improved salt tolerance

**Figure 6:**
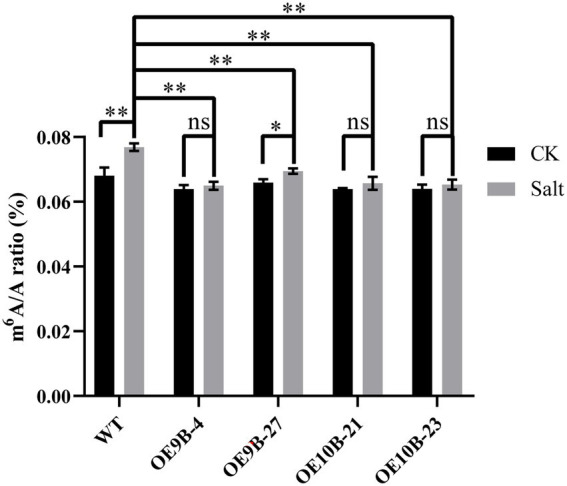
Global N^6^-methyladenosine (m^6^A) RNA methylation level in WT and transgenic lines under normal conditions and salt treatment. The data represented averages ± SE of three replicates. The different letters indicated significant differences between different tissue (*p  < 0.05, **p  < 0.01).

**Figure 7:**
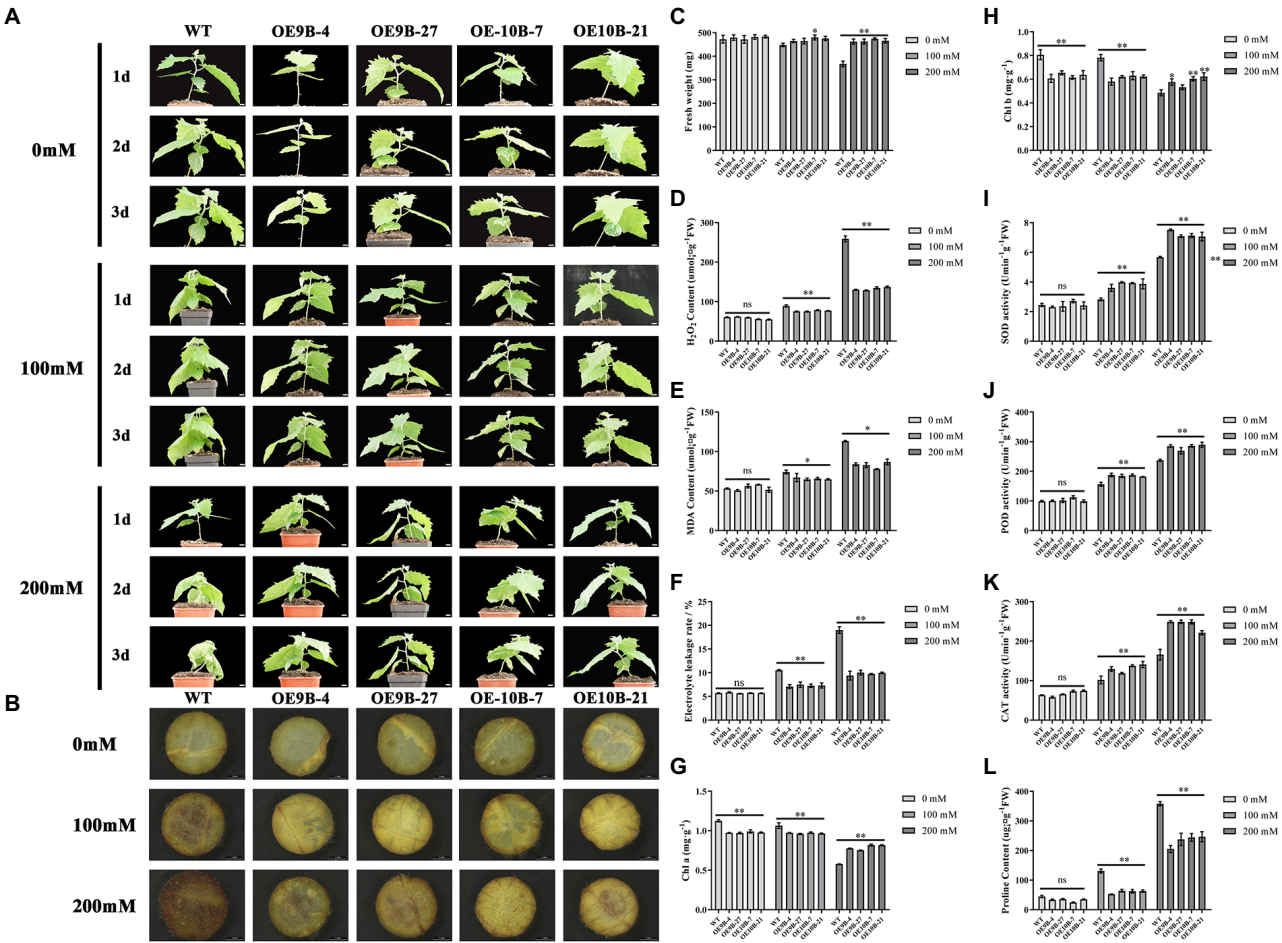
Overexpressing *PagALKBH9B* and *PagALKBH10B* enhanced salt tolerance in transgenic poplars. **(A)** The phenotype of transgenic lines and WT under 0, 100, and 200 mM NaCl treatment. Bar = 1 cm. **(B)** Histochemical detection of H_2_O_2_ accumulation of WT and transgenic lines under a certain concentration salt treatment by DAB staining. **(C)** The fresh weight of leaves of WT and transgenic lines under a certain concentration salt treatment. **(D)** Histochemical detection of H_2_O_2_ accumulation of WT and transgenic lines under a certain concentration salt treatment. **(E)** Histochemical detection of MDA contents of WT and transgenic lines under a certain concentration salt treatment. **(F)** The electrolyte leakage rates of WT and transgenic lines under a certain concentration salt treatment. **(G)** The Chl content of WT and transgenic lines under a certain concentration salt treatment. **(H)** The Chl b content of WT and transgenic lines under a certain concentration salt treatment. **(I–K)** Antioxidant enzyme activity in transgenic lines compared with WT after salt treatment, respectively. **(L)** Histochemical detection of proline contents of WT and transgenic lines under a certain concentration salt treatment. The experiments were performed three times and the similar results were obtained, at least six 84K poplars were collected as a replicate. The bars represent means ± SE (*n* = 6) and the asterisks indicate significant differences between WT and transgenic plants (*p < 0.05, **p < 0.01).

m^6^A methyltransferase can improve salt tolerance in *Arabidopsis* by increasing the overall level of m^6^A methylation modification (Hu et al., 2021). We speculated whether *PagALKBH9B* and *PagALKBH10B* had the opposite function in response to salt stress in poplar. We first analyzed the overall level of m^6^A between different lines under salt stress, and found that salt stress could significantly induce higher up-regulation of m^6^A levels in WT (Figure 6). To determine the role of *PagALKBH9B* and *PagALKBH10B* in response to salt stress in poplar, we examined the phenotypic changes of overexpressing transgenic lines and WT under different concentrations of salt treatment. As shown in Figure 7A, there was no visible morphological difference between transgenic lines and WT under control (0 mM). When exposed to different concentrations of salt (100 and 200 mM), the WT rapidly showed different degrees of pressure injury with leaf wilting and fresh weight decreased (Figure 7C), while the transgenic lines were less sensitive to salt stress than WT plants. These results indicated that overexpression of *PagALKBH9B* and *PagALKBH10B* in poplar alleviated the phenotypic injury induced by salt stress.

Salt stress can lead to the accumulation of reactive oxygen species (ROS) in plant leaves, which is an important factor that cause oxidative damage in plants (Yu et al., 2020). Therefore, we analyzed the H_2_O_2_ content of each line treated with different concentrations (Figures 7B,D). Under normal conditions, there was no significant difference in H_2_O_2_ content between WT and overexpression lines. However, after different concentrations of salt treatment, discs of *PagALKBH9B* and *PagALKBH10B* -overexpressing lines exhibited lighter and less intense brown patterns than those of the WT, the accumulation of H_2_O_2_ in WT was significantly higher than that in transgenic lines. This indicated that overexpression of *PagALKBH9B* and *PagALKBH10B* both could reduce the accumulation of ROS under salt stress.

Under salt stress, the accumulation of ROS in plants can cause or aggravate membrane lipid peroxidation, resulting in cell membrane system damage and even cell death. MDA is one of the products of membrane lipid peroxidation and an important indicator of the degree of membrane lipid peroxidation, which can indirectly reflect the degree of membrane system damage and plant stress resistance (Yu et al., 2020). As shown in Figure 7E, there was no significant difference in MDA content between overexpression transgenic lines and WT under normal conditions. After stress treatment, the MDA content in both plants increased, but the increase of over-expression transgenic plants was significantly lower than that of WT, and the results were consistent in different concentrations of salt treatment. After salt treatment, the electrolyte leakage rate gradually increased with treatment concentration. Although there was no significant difference in electrolyte leakage rate between WT and transgenic lines under normal conditions, the electrolyte leakage rate of WT was significantly higher than that of transgenic lines after salt stress treatment (Figure 7F). The above results showed that the overexpression of *PagALKBH9B* and *PagALKBH10B* reduced the membrane lipid peroxidation caused by salt stress.

Salt stress can affect plant photosynthesis by reducing Chl a/b (Tang C. et al., 2018), therefore, we compared the Chl a/b content of WT and transgenic lines (Figures 7G,H). Although WT contained more Chl a/b than transgenic lines under normal conditions, the content of Chl a/b in WT decreased significantly with the increase of salt concentration. Finally, the content of Chl a/b in WT was significantly lower than that in transgenic lines at 200 mM salt concentration.

In order to further evaluate the oxidative damage of plants after salt stress, we measured the protective enzyme activities of transgenic lines and WT after salt stress, including SOD, POD, and CAT activities. Under normal growth conditions, there was no significant difference in SOD, POD, and CAT activities between WT and overexpression transgenic plants (Figures 7I–K). However, after salt stress, the activities of SOD, POD, and CAT in WT and overexpression transgenic plants were significantly increased. The contents of SOD, POD, and CAT in WT and transgenic lines treated with 100 and 200 mM NaCl were significantly higher than those in WT. The expression abundance detection of SOD, POD, and CAT synthetic regulatory-related genes (*Cu/Zn-SOD*, *POD-20,* and *CAT*) had higher expression in transgenic lines, but there was no significant difference in m^6^A modification level (Supplementary Figure S6). Overexpression of *PagALKBH9B* and *PagALKBH10B* increased the activities of SOD, POD, and CAT in transgenic plants, enhanced the scavenging ability of reactive oxygen species, and reduced oxidative damage.

Salt stress can lead to osmotic stress, and proline is an important osmotic adjustment substance, which is up-regulated after stress, so its content reflects plant stress resistance (Yu et al., 2020). There was no significant difference in proline content between transgenic lines and WT under control. However, with the increase of salt concentration, the proline content increased, but at all salt concentrations, the proline content in overexpression transgenic plants was significantly lower than that in WT (Figure 7L). *P5C5* has been shown to regulate proline synthesis (Kubala et al., 2015) and the expression abundance of *P5C5* lower in overexpression transgenic plants and m^6^A enrichment level was no significant difference (Supplementary Figure S6). Proline is a key marker for drought stress response in plants (Chen et al., 2022), so we compared the drought resistance between WT and transgenic lines. The results showed that overexpression of *PagALKBH9B* and *PagALKBH10B* increased the sensitivity of transgenic lines to drought stress (Supplementary Figure S7A). Consistent with the proline content under salt stress, the proline content of transgenic lines was lower than WT under drought stress (Supplementary Figure S7B).

## Discussion

RNA demethylation mediated by ALKBHs acting as eraser proteins was considered to be an important part of the epigenetic regulatory network for plant growth, development, and abiotic stress responses (Huong et al., 2020; Liang et al., 2020). Yet, the molecular mechanisms of ALKBH regulation need to be further explored, especially in woody plants. Unlike the known *AtALKHs* and *OsALKBHs* (Liang et al., 2020), more *PagALKBHs* were identified from the poplar 84K hybrid (*Populus alba* × *P. tremula* var. *glandulosa*) by comparison of the alignments of homologous ALKBH protein sequences in *A. thaliana* (Liang et al., 2020). Function is often conserved between paralogs (Shao et al., 2019; Cheng et al., 2021). Sequence alignments between homologs of *PagALKBHs* (Figure 1A; Supplementary Figure S1) showed high similarity and high collinearity (Figure 1B). Orthologous proteins generally perform analogous biological functions in diverse species within the genus (Schlicker et al., 2006).

Homologs of *PagALKBHs* contain similar exon/intron structure and conserved motifs (Figures 1C,D), thus, the homologs of *PagALKBH* between hybrid parents may have functional similarity. The catalytic activity of ALKBH demethylase depends on Fe^2+^ (Falnes et al., 2002; Trewick et al., 2002; Fedeles et al., 2015) and all PagALKBHs contained an Fe^2+^ binding domain (Figure 1C) so that *PagALKBHs* may mediate in the oxidative demethylation of nucleic acids. Although there were homologous catalytic centers in the conserved sequences, substrate selectivity was characteristic of the ALKBH family (Yi et al., 2009; Ougland et al., 2015). Phylogenetic analysis was a useful way to identify functional orthologous proteins (Bauwens et al., 2018). The ALKBHs were divided into six phylogenetic groups, consistent with previous studies (Figure 2). Among the groups, ALKBH9 and ALKBH10 were homologs of human demethylase ALKBH5 (Tang Y. et al., 2018), which can remove m^6^A methylation in *Arabidopsis,* and the ALKBH9 group was shown to have functional redundancy (Duan et al., 2018). Similar to the results from other plants, the human demethylase FTO (fat mass and obesity-associated protein) also has no homologs in poplar (Chen et al., 2012).

N^6^-methyladenosine (m^6^A) RNA methylation was the most abundant chemical modification in eukaryotic cells, accounting for 80% of RNA base modifications (Kierzek, 2003). RNA methylation is a dynamic process and requires the participation of methyltransferases (writers), demethylation (erasers), and recognition proteins (readers). MTA, MTB, FIP37, VIRILIZER, and HAKAI were considered to be the main components of the m^6^A methyltransferase complexes in plants (Bodi et al., 2012; Ra et al., 2017; Yue et al., 2019). The m^6^A readers were members of the YTH family, and the members YTHDF1, YTHDF2, and YTHDF3 recognize m^6^A modification sites (Dominissini et al., 2012; Wang et al., 2015). In mammals, the first identified m^6^A demethylase was FTO. Later, ALKBH5 (alkylation repair homolog 5) was shown to be a mRNA m^6^A demethylase (Jia et al., 2011; Mauer et al., 2017). ALKBH9B and ALKBH10B are homologs of human demethylase ALKBH5 in plants (Figure 2). m^6^A can revert to adenosine through the action of ALKBH9B and ALKBH10B (Martínez-Pérez et al., 2017; Duan et al., 2018). The human ALKBH5 homologs of PagALKBH9B and PagALKBH10B were also confirmed to regulate the global level of m^6^A RNA methylation. Overexpression of *PagALKBH9B* and *PagALKBH10B* reduced the global m^6^A RNA methylation in transgenic plants (Figure 4A). However, the m^6^A methylation level on mRNA of transgenic lines overexpressing *PagALKBH9B* did not change significantly (Figure 4B). Martínez-Pérez et al. (2017) reported that ALKBH9B can remove m^6^A from single-stranded RNA (ssRNA) *in vitro*, the analysis of Duan et al. (2018) showed that the methylation level of m^6^A of mRNA in *alkbh9b* mutant did not change significantly in its highly expressed organs. It is possible that ALKBH9B was not involved in the removal of m^6^A modifications on mRNA. Considering that *PagALKBH9B* and *PagALKBH10B* transgenics were driven by CaMV35S, the activity of the overexpressed enzymes may increase demethylated RNAs or demethylate RNAs that were not normally recognized by these proteins, which may lead to novel phenotypes in the overexpressed lines.

m^6^A RNA methylation plays a crucial and dynamic role in many processes, including development of organs (Vespa et al., 2004; Bodi et al., 2012; Chen et al., 2018a,b; Arribas-Hernández and Brodersen, 2020), seed germination (Zhong et al., 2008) and fruit maturation (Zhou et al., 2019, 2021). The homologs ALKBH9 and ALKBH10 were shown to regulate fruit maturation in tomatoes (Zhou et al., 2019), and the floral transition process in *A. thaliana* (Duan et al., 2018). Overexpression of *PagALKBH9B* and *PagALKBH10B* inhibited the formation of ARs and reduced biomass accumulation of transgenic lines. The transcriptional abundance of the genes involved in the regulation of adventitious roots was inhibited in the overexpression lines (Sorin et al., 2006; Kim et al., 2007; Sukumar et al., 2013), but their m^6^A enrichment levels were not significantly different between different lines (Supplementary Figure S3). Perhaps there was no m^6^A modification in their transcription mRNA, and they were regulated by the indirect inhibition of *PagALKBH9B* and *PagALKBH10B*, thereby inhibiting the formation of adventitious roots. Plant height was also inhibited by overexpression of *PagALKBH10B* transgenic lines (Figure 5; Supplementary Figure S4). The result may depend on their regulation of global m^6^A RNA methylation. Transgenic expression is highly variable and such variation may be the basis for the difference between the expression of *PagALKBH9B* and *PagALKBH10B*. Whether ALKBH9B and ALKBH10B have the same target RNA, remains to be determined. Overexpression of m^6^A RNA methyl transferase (MTA) increased m^6^A RNA methylation, which could result in a more elaborate root system (Lu et al., 2020). The formation of ARs is a complex process, in which RNA m^6^A modification sites and their roles should be studied further using high-throughput sequencing.

High salt stress is one of the main abiotic stresses, plants evolved a series of adaptive physiological mechanisms, such as the synthesis of osmotic adjustment substances, the regulation of ion balance *in vivo*, and the removal of excessive accumulation of reactive oxygen species (Zhao et al., 2020). m^6^A methylation of RNA has also been shown to be involved in plant response to salt stress. By mapping m^6^A modification in two sorghum genotypes (salt-tolerant M-81E and salt-sensitive Roma) with different salt tolerance, it is indicated that the quantity and extent of m^6^A modification in salt-tolerant gene transcripts may be an important factor in salt tolerance (Zheng et al., 2021). Previous studies have confirmed that m^6^A methyltransferase mutants, including *mta*, *mtb*, *virilizer,* and *hakai*, display salt-sensitive phenotypes in m^6^A-dependent manner. VIR-mediated m^6^A methylation regulates the homeostasis of reactive oxygen species by negatively regulating the mRNA stability of several negative regulatory factors *ATAF1*, *GI,* and *GSTU17* under salt stress (Hu et al., 2021). In the present study, we found that salt stress induced upregulation of total RNA m^6^A level, whereas overexpression of *PagALKBH9B* and *PagALKBH10* partially inhibited salt stress-induced m^6^A level (Figure 6). In addition, salt treatment activated the expression of *PagALKBH9B* and *PagALKBH10B* (Supplementary Figure S5). This indicates that m^6^A methylation in poplar may also be involved in the response to salt stress.

The ALKBH gene family has been shown to be widely involved in salt stress regulation. Under salt stress, the survival rate of *alkbh6* mutant was significantly lower than that of wild type (Huong et al., 2020), overexpression of *ALKBH8B* improved the salt tolerance of transgenic *Arabidopsis* plants (Huong et al., 2022). In *Arabidopsis*, *ALKBH10B* can regulate salt stress resistance and ABA signaling by mediating m^6^A modification levels of *ATAF1*, *BGLU22*, *MYB73*, *ABI3,* and *ABI4*, germination of *alkbh10b* mutant seeds was significantly delayed and seedling growth and survival rate were enhanced under salt stress (Shoaib et al., 2021). In our present study, we found that overexpression of *PagALKBH9B* and *PagALKBH10B* increased salt tolerance (Figure 7A). Further comparison of WT and transgenic lines confirmed that *PagALKBH9B* and *PagALKBH10B* could reduce salt stress-induced H_2_O_2_ accumulation and oxidative damage by increasing the activities of antioxidant enzyme systems SOD, POD, and CAT, and reduce the damage of salt stress on chloroplasts (Figures 7B–K). The expression abundance of antioxidant enzyme synthesis genes in transgenic lines was higher, however, we did not detect differences in the m^6^A enrichment level of mRNA between different lines (Supplementary Figure S6), they may not be the direct target genes of PagALKBH9B and PagALKBH10B. Overexpression of *PagALKBH9B* and *PagALKBH10B* increased sensitivity to drought stress by inhibiting proline synthesis (Figure 7L; Supplementary Figure S7), although the expression of proline synthesis gene *P5C5* decreased, the level of m^6^A enrichment did not change significantly (Supplementary Figure S6). Discovery of direct target genes for PagALKBH9B and PagALKBH10B may depend on sequencing technologies such as MeRIP-seq.

m^6^A methyltransferase and demethylase regulatory genes have the same function that has been confirmed in fruit ripening. Mutation of *SlALKBH2* (the *ALKBH9B* and *ALKBH10B* homologous gene) reduced the mRNA abundance of *SlDML2* and delays fruit ripening (Zhou et al., 2019). MTA and MTB can regulate fruit ripening by mediating the mRNA stability and translation efficiency of ABA pathway genes, overexpression of *MTA* and *MTB* will promote fruit ripening, while gene silencing of *MTA* and *MTB* will delay fruit ripening (Zhou et al., 2021). The reason may be that there is a certain deviation between the known target genes of methyltransferase and demethylase, and no research has yet proved that methyltransferase and demethylase of a target gene were known. In the current study, we found that overexpression of *PagALKBH9B* and *PagALKBH10B* could inhibit ARs formation (Figure 5) and improve salt tolerance (Figure 7), which was closely related to their homologous genes of ALKBH5 (Figure 2) and their function of reducing total RNA m^6^A methylation (Figure 4A). However, the difference in domain determined that their functions have certain specificity, *PagALKBH9B* was not involved in the regulation of m^6^A methylation on mRNA (Figure 4B) and had no significant effect on *IAA28* expression (Supplementary Figure S3) and plant height (Supplementary Figure S4). In addition, the relationship between them in regulating RNA m^6^A methylation needs to be further determined. Although we preliminarily verified the similarity between *PagALKBH9B* and *PagALKBH10B* in regulating adventitious root formation and salt tolerance, whether the target genes of the two demethylases are consistent needs to be further explored by MeRIP-seq and other technologies.

## Conclusion

We identified the PagALKBH family of poplar 84K, analyzed its sequences, and studied its role in growth and development and response to salt stress. Twenty-three *PagALKBHs* were identified and similar amino acid sequence, gene structure, and conserved domains were determined between homologs. Due to high conservation of *PagALKBH,* the homologs of poplar 84K can be divided into six groups based on their phylogeny. Expression analysis revealed the tissue specificity for leaves of most of the *PagALKBHs*, *PagALKBH10C* was the exception. Overexpression of *PagALKBH9B* and *PagALKBH10B* both inhibited adventitious root formation and reduced biomass accumulation of transgenic plants by reducing the m^6^A RNA methylation, furthermore, *PagALKBH10B* can also negatively regulate plant height. Overexpression of *PagALKBH9B* and *PagALKBH10B* can reduce the accumulation of H_2_O_2_ and oxidative damage by increasing the activities of SOD, POD, and CAT, and reducing the damage of salt stress on chloroplasts. But overexpression line reduced the content of proline, thereby increased sensitivity to drought stress. This research helped to better understand the biological functions of ALKBHs and m^6^A RNA methylation in *Populus* and suggests new candidate genes and feasible strategies for tree species improvement.

## Data availability statement

The original contributions presented in the study are included in the article/Supplementary material, further inquiries can be directed to the corresponding author.

## Author contributions

YZ and YL designed the experiments and wrote the manuscript. YZ, QG, SC, YT, and KH performed the experiments. YZ and YS analyzed the data. JL, QY, and QJ provided guidelines for the research. RS provided advice and amendments to the manuscript. All authors contributed to the article and approved the submitted version.

## Funding

This work was supported by the Fundamental Research Funds for the Central Universities (2015ZCQ-SW-03), the Major National Science and Technology Projects (2018ZX08020002-003-002), and the National Science Foundation of China (31971675).

## Conflict of interest

The authors declare that the research was conducted in the absence of any commercial or financial relationships that could be construed as a potential conflict of interest.

## Publisher’s note

All claims expressed in this article are solely those of the authors and do not necessarily represent those of their affiliated organizations, or those of the publisher, the editors and the reviewers. Any product that may be evaluated in this article, or claim that may be made by its manufacturer, is not guaranteed or endorsed by the publisher.

## Footnotes

^1^ http://plants.ensembl.org/index.html

^2^ https://phytozome.jgi.doe.gov/pz/portal.html

^3^ http://pfam.xfam.org/

^4^ http://smart.embl-heidelberg.de/

^5^ http://gsds.cbi.pku.edu.cn/

^6^ https://meme-suite.org/meme/
