# Supplementary figures and images for: Genome-wide identification of the AlkB homologs gene family, PagALKBH9B and PagALKBH10B regulated salt stress response in Populus
Source: Front Plant Sci. 2022 Sep 20;13:994154. doi: 10.3389/fpls.2022.994154 (PMC9530910; doi:10.3389/fpls.2022.994154)

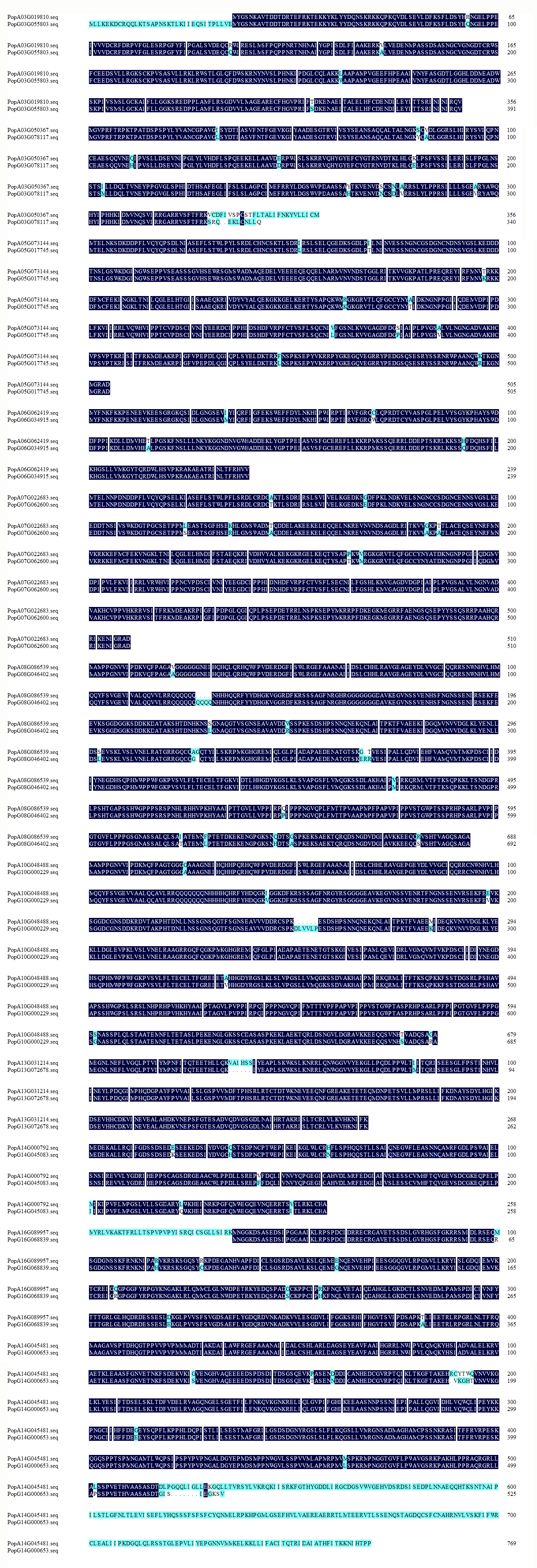

Supplement: Supplementary file 1 [file Image_1.TIF]

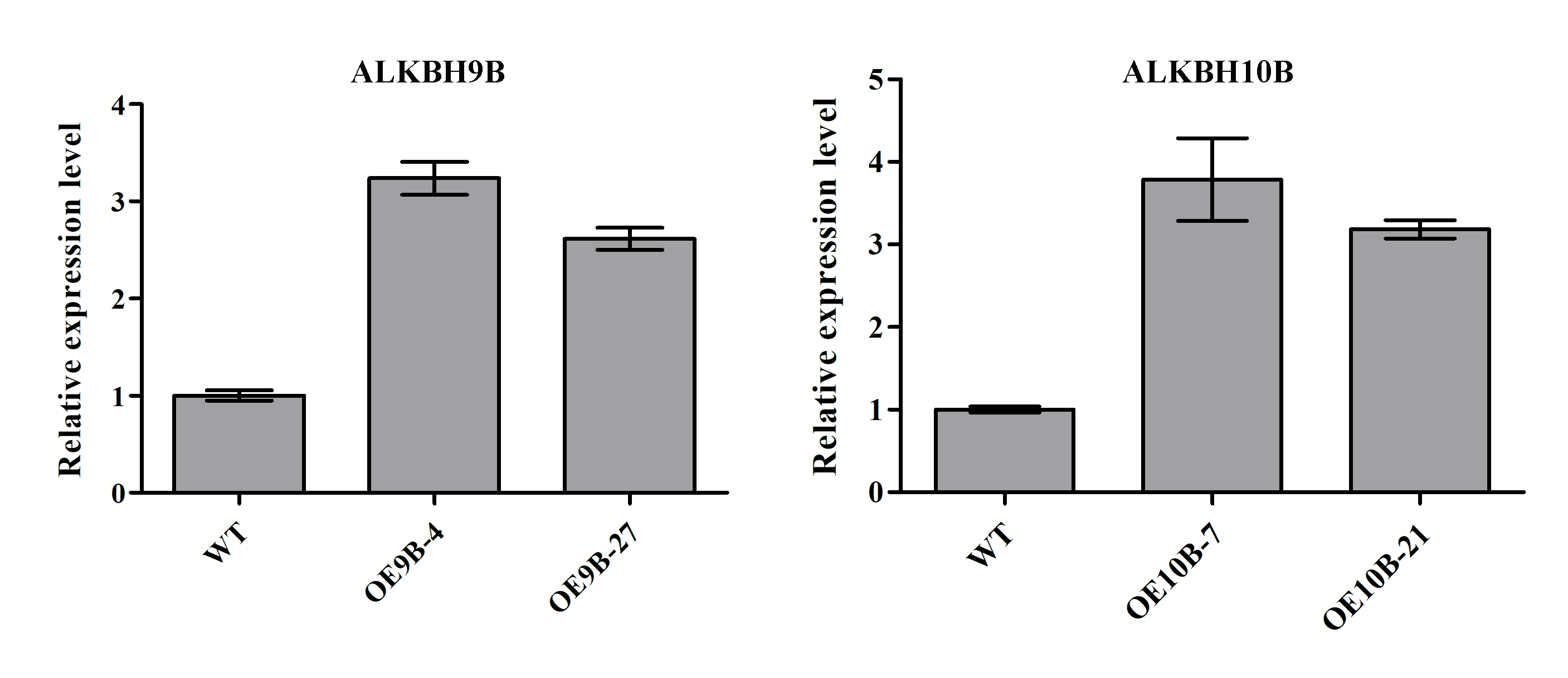

Supplement: Supplementary file 2 [file Image_2.TIF]

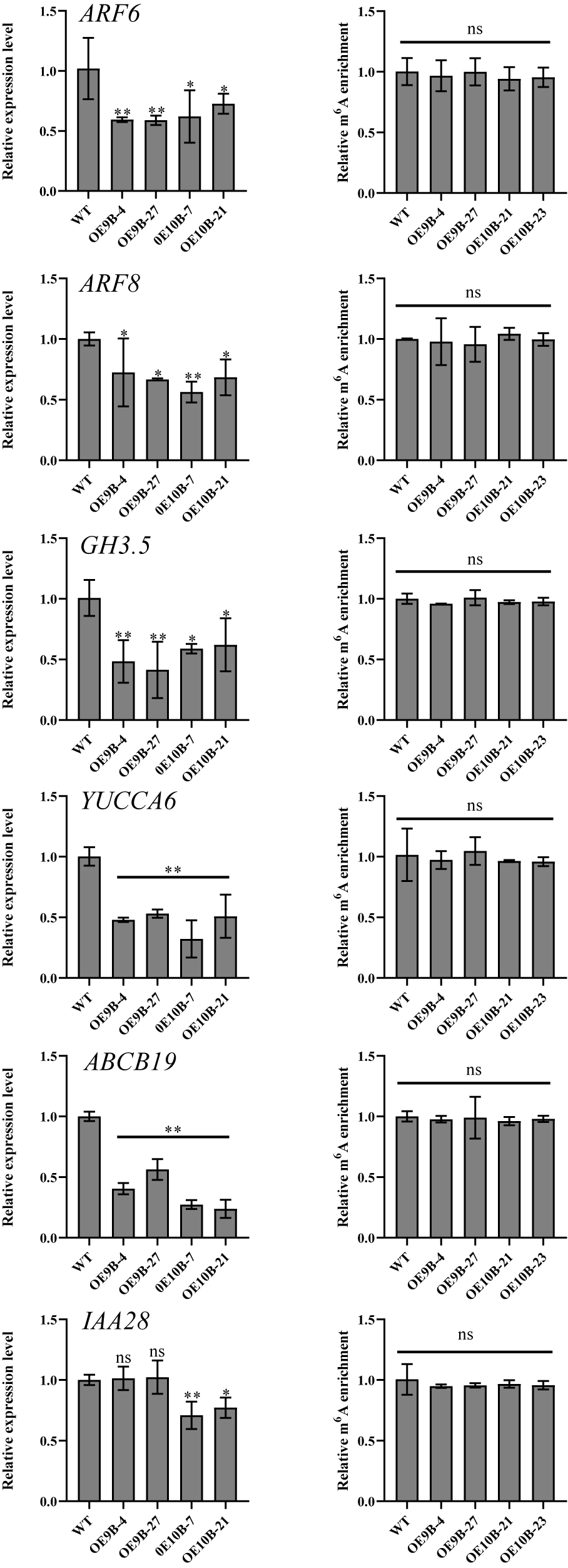

Supplement: Supplementary file 3 [file Image_3.TIF]

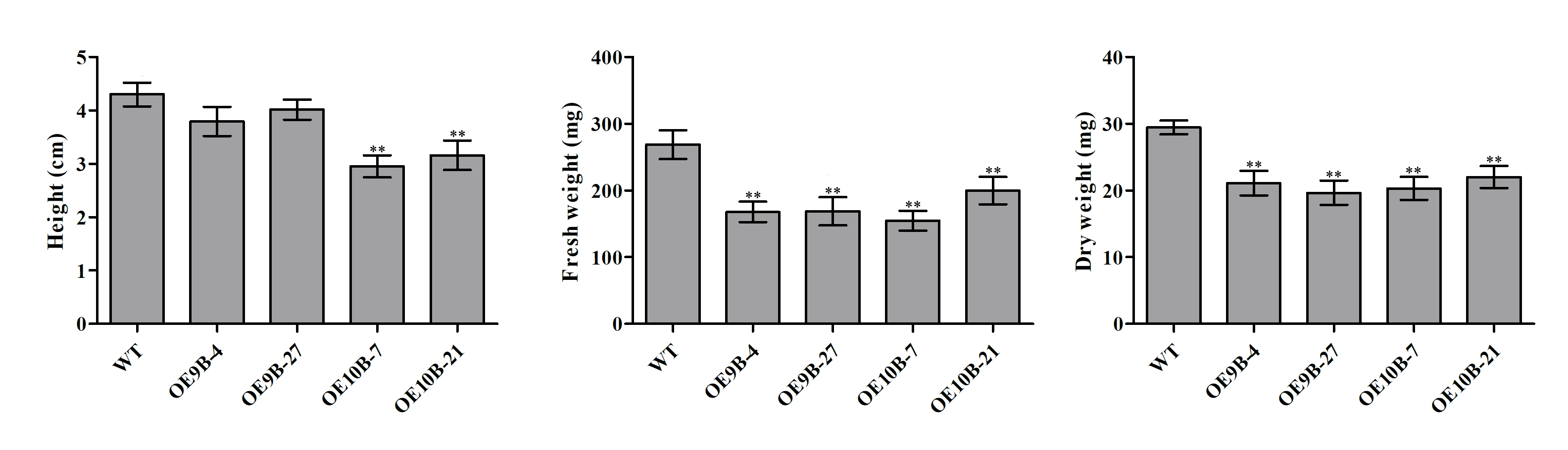

Supplement: Supplementary file 4 [file Image_4.TIF]

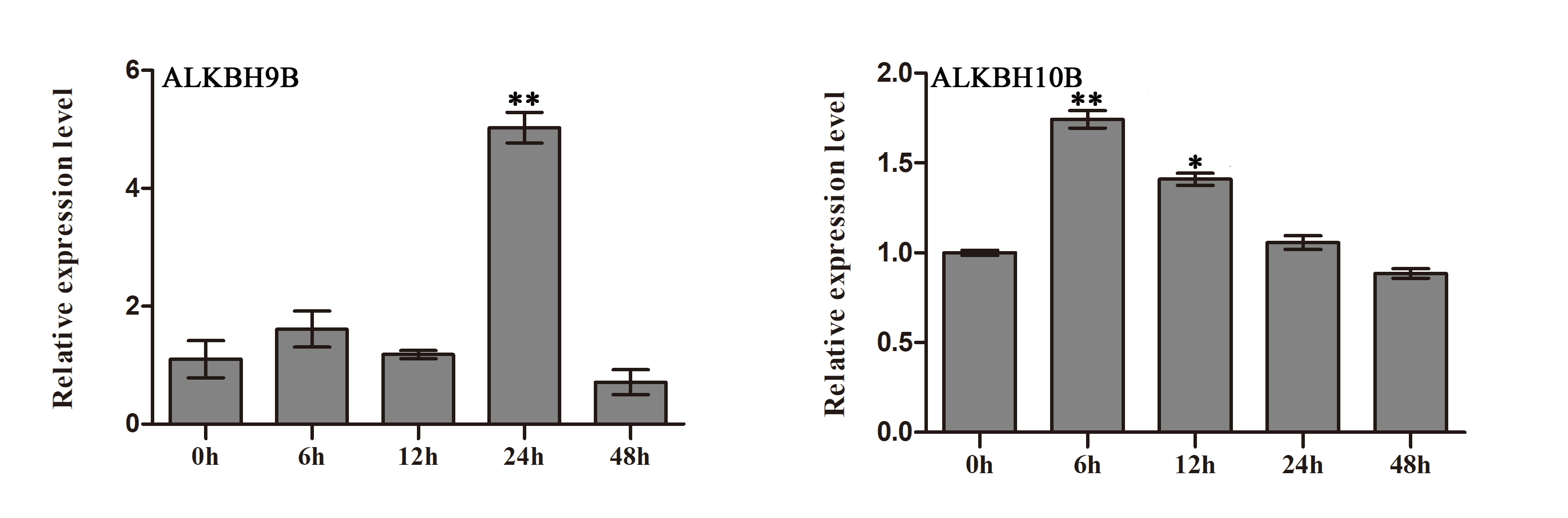

Supplement: Supplementary file 5 [file Image_5.jpg]

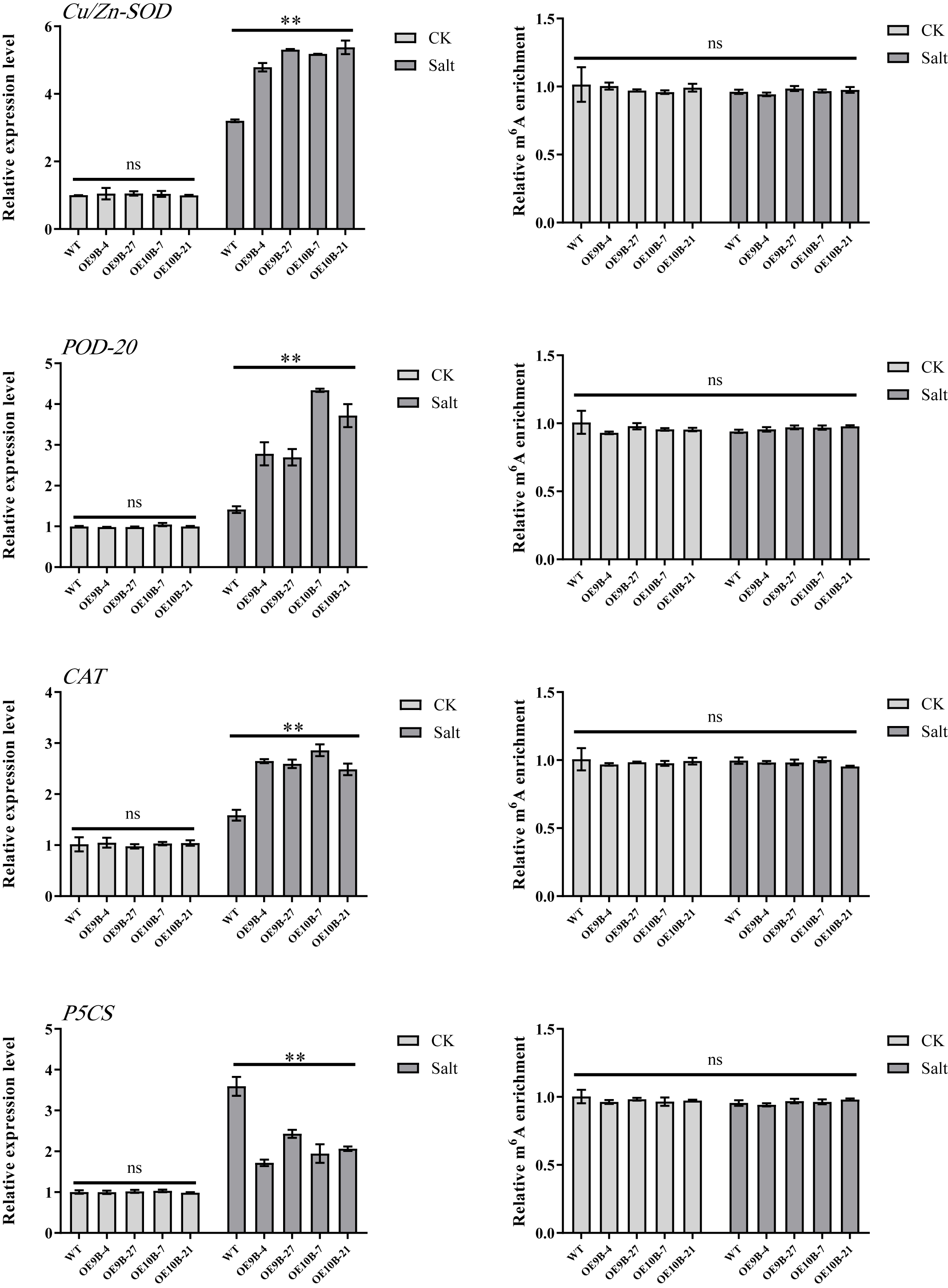

Supplement: Supplementary file 6 [file Image_6.jpg]

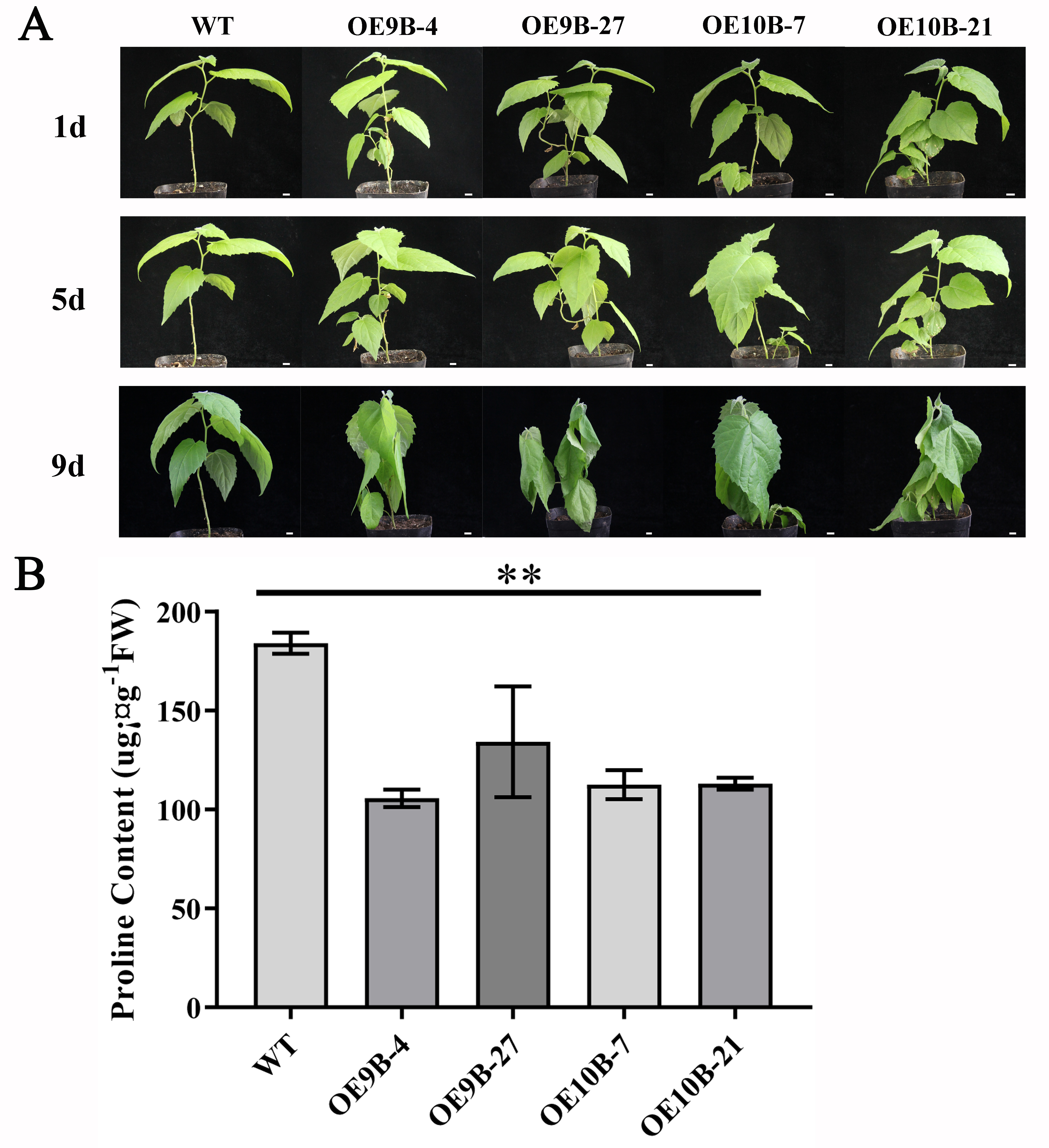

Supplement: Supplementary file 7 [file Image_7.jpg]
