# Supplementary material for: Genome-wide identification of the AlkB homologs gene family, PagALKBH9B and PagALKBH10B regulated salt stress response in Populus
Source: Front Plant Sci. 2022 Sep 20;13:994154. doi: 10.3389/fpls.2022.994154 (PMC9530910; doi:10.3389/fpls.2022.994154)
Supplement: Supplementary file 8 [file Table_1.DOCX]

Supplementary Table S1. Primers used in the article.

| Primer names | Primer sequences (5’ - 3’) |
| --- | --- |
| *Actin*-F | AAGATTCCGTTGTCCAGAGGTCCT |
| *Actin*-R | GAACATAGTAGAGCCACCACTGAGAAC |
| *ALKBH1A*-F | TCCAATAAAGCCGTTACCG |
| *ALKBH1A*-R | TCAGGAGGAAGTTCACCGT |
| *ALKBH1B*-F | CTGGGAGTAGAGATTCTGCTG |
| *ALKBH1B*-R | GGCAAGTCTTCACCATTTCA |
| *ALKBH1C*-F | GCAACAGAGACAGAAACTCG |
| *ALKBH1C*-R | CTGACTGATTTGACCCAAGAC |
| *ALKBH2*-F | AAGTCCAGGATTGCCAGA |
| *ALKBH2*-R | CAACATTGTCATTGCCACC |
| *ALKBH6*-F | TCAAGGACAATGCCTACTCA |
| *ALKBH6*-R | ATCTCCACTTCCCACATCTT |
| *ALKBH8A*-F | GGACCAACTCACAGTAAACGA |
| *ALKBH8A*-R | TAGAGGCAGCATCAGGAAC |
| *ALKBH8B*-F | ATCTTTGGCGACTCATCAG |
| *ALKBH8B*-R | ATAGCGGAGAGGAGGGTAGA |
| *ALKBH9A*-F | TCCGATAAAGATGACCCG |
| *ALKBH9A*-R | CTTGGAGTTCTGAGAGAGAGC |
| *ALKBH9B*-F | ATAATCCCGATAACGATGACCC |
| *ALKBH9B*-R | ACTGAGAGAGAGCGAATGCG |
| *ALKBH10A*-F | CCAGGAAATGTGGTCATACC |
| *ALKBH10A*-R | TGGTGGCAGAGAGAATCAA |
| *ALKBH10B*-F | AGGATTCACAGCCAAGGAG |
| *ALKBH10B*-R | TCGCCTGAGAGTTCACCAT |
| *ALKBH10C*-F | GCGATTATTGACTCCCTCTG |
| *ALKBH10C*-R | TTGCTGCTGTCTCCTCAGT |
| 35S-F | GACGCACAATCCCACTATCC |
| *ALKBH9B*-F | GAGAACACGGGGGACTCTAGAATGACCGAACTCAATAAATCCGA |
| *ALKBH9B*-R | CGATCGGGGAAATTCGAGCTCTCATCCATCAGCTCTGCCCA |
| *ALKBH10B*-F | GAGAACACGGGGGACTCTAGAATGGCGGCAGGAGCAGTAT |
| *ALKBH10B*-R | CGATCGGGGAAATTCGAGCTCTCAGACCGATTTCCCTTCAATAC |
| *ARF6*-F | GCATCAACGAACAAGGAAG |
| *ARF6*-R | ATCTGCTGGAAGGTAGGCA |
| *ARF8*-F | ATTACAGCCTTTGACACCG |
| *ARF8*-R | AAGAGACTACCAGTGGAGGAAA |
| *GH3.5*-F | CAACTGCGGTGTGGAGTAT |
| *GH3.5*-R | GGATAGAAGTGTTATGGGCTG |
| *YUCCA6*-F | TTACAACCTCGCTGAGAAGA |
| *YUCCA6*-R | TAATCACAGGCACGGCTT |
| *ABCB19*-F | GGTATTGTTGTGTGCCTATCG |
| *ABCB19*-R | CAAGAAGGGTGTCAGTTGAGAC |
| *IAA28*-F | CATTGATGGGTCTACTGGG |
| *IAA28*-R | CCTACCACCTGTGTCTTTGC |
| *Cu/Zn-SOD*-F | ACAACCAGATTCCTCTTACT |
| *Cu/Zn-SOD*-R | CAATAACACCACATGCTACT |
| *POD-20*-F | TCTTGACATAGGAGACTTGG |
| *POD-20*-R | ATGGACCGTAGGATTCTTC |
| *CAT*-F | GGATGAGGAGGTCAACTATT |
| *CAT*-R | AATCGTTCTTGCCTGTC |
| *P5CS*-F | CGAAGTTATCGTGGTGACAT |
| *P5CS*-R | TCTTGAAATCCCTATCGGTC |
