# Supplementary material for: Genome-wide identification of the AlkB homologs gene family, PagALKBH9B and PagALKBH10B regulated salt stress response in Populus
Source: Front Plant Sci. 2022 Sep 20;13:994154. doi: 10.3389/fpls.2022.994154 (PMC9530910; doi:10.3389/fpls.2022.994154)
Supplement: Supplementary file 9 [file Table_2.DOCX]

Supplementary Table S2: List of the *PagALKBH* genes identified in poplar 84K

|  | *Arabidopsis thaliana* | *Oryza sativa* | Poplar 84K | |
| --- | --- | --- | --- | --- |
|  |  |  | *Populus alba* | *Populus tremula* var*. glandulosa* |
| ALKBH1 | AT1G11780 | Os03g0816500 | PopA03G019810 | PopG03G055803 |
|  | AT3G14140 |  | PopA16G089957 | PopG03G011269 |
|  | AT3G14160 |  |  | PopG16G068839 |
| ALKBH2 | AT2G22260 | Os06g0286310 | PopA06G062419 | PopG06G034915 |
| ALKBH6 | AT4G20350 | Os10g0420000 | PopA13G031214 | PopG13G072678 |
| ALKBH8 | AT1G31600 | Os04g0602700 | PopA03G050367 | PopG03G078117 |
|  | AT4G02485 | Os11g0657200 | PopA14G000792 | PopG14G045083 |
| ALKBH9 | AT4G36090 | Os11g0657200 | PopA05G073144 | PopG05G017745 |
|  | AT2G17970 |  | PopA07G022683 | PopG07G062600 |
|  | AT1G48980 |  |  |  |
| ALKBH10 | AT1G14710 | Os03g0238800 | PopA10G048488 | PopG10G000229 |
|  | AT2G48080 | Os10g0116900 | PopA08G086539 | PopG08G046402 |
|  | AT4G02940 | Os05g0401500 | PopA14G045481 | PopG14G000653 |
